# A Study on Arrhythmia via ECG Signal Classification Using the Convolutional Neural Network

**DOI:** 10.3389/fncom.2020.564015

**Published:** 2021-01-05

**Authors:** Mengze Wu, Yongdi Lu, Wenli Yang, Shen Yuong Wong

**Affiliations:** ^1^Department of Information Engineering, Wuhan University of Technology, Wuhan, China; ^2^Department of Electrical and Electronics Engineering, Xiamen University Malaysia, Sepang, Malaysia; ^3^Department of Electrical and Automation Engineering, Nanjing Normal University, Nanjing, China

**Keywords:** deep learning, ECG, anti-noise performance, feature classification, convolutional neural network

## Abstract

Cardiovascular diseases (CVDs) are the leading cause of death today. The current identification method of the diseases is analyzing the Electrocardiogram (ECG), which is a medical monitoring technology recording cardiac activity. Unfortunately, looking for experts to analyze a large amount of ECG data consumes too many medical resources. Therefore, the method of identifying ECG characteristics based on machine learning has gradually become prevalent. However, there are some drawbacks to these typical methods, requiring manual feature recognition, complex models, and long training time. This paper proposes a robust and efficient 12-layer deep one-dimensional convolutional neural network on classifying the five micro-classes of heartbeat types in the MIT- BIH Arrhythmia database. The five types of heartbeat features are classified, and wavelet self-adaptive threshold denoising method is used in the experiments. Compared with BP neural network, random forest, and other CNN networks, the results show that the model proposed in this paper has better performance in accuracy, sensitivity, robustness, and anti-noise capability. Its accurate classification effectively saves medical resources, which has a positive effect on clinical practice.

**Graphical Abstract:**
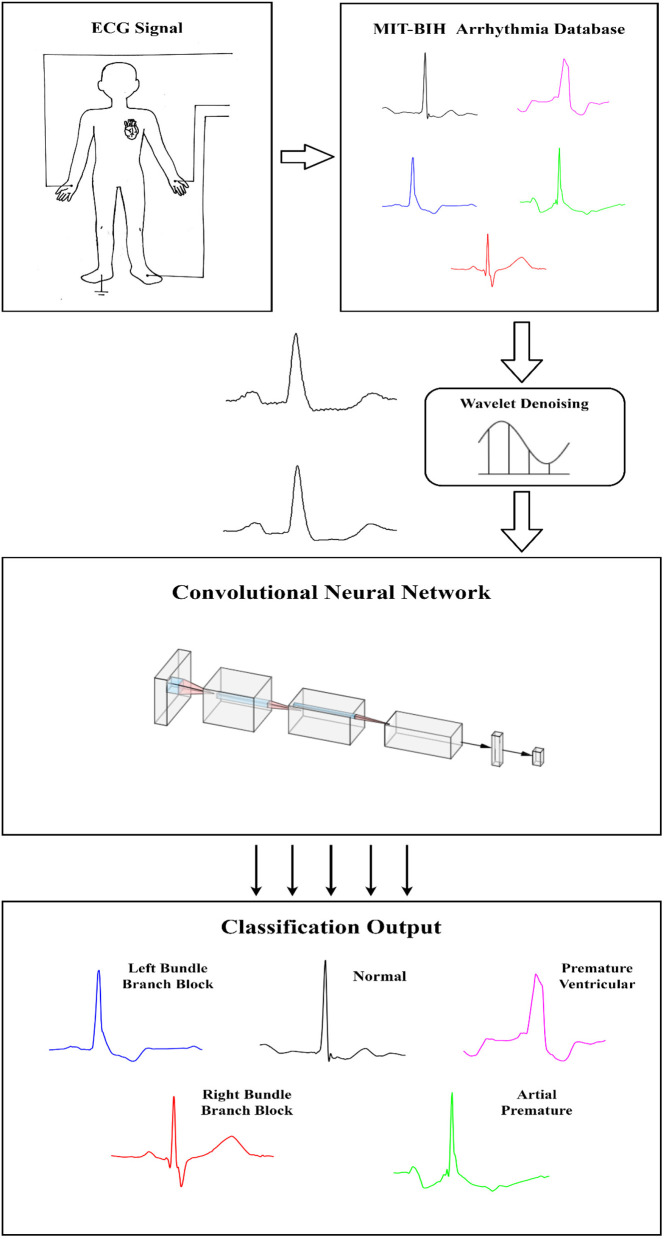
The graphical abstract of the methodology.

## Introduction

Cardiovascular disease is a common disease that seriously threatens human health, especially the health of middle-aged and older people. It is characterized by high prevalence, high disability, and high mortality. Nowadays, the world is facing with the aging population. The increasing aggravation of cardiovascular disease has become a major public health problem (Mc Namara et al., 2019). ECG analysis is an effective way of evaluating heart health. Therefore, the identification and classification of ECG signals are essential to cardiovascular diseases. Not only for early prevention but also necessary for timely detection and proper treatment. It is of considerable significance to study the classification of related ECG signals (Guo et al., 2016; Yin et al., 2016).

The electrocardiogram is a visual time series that records the electrical activity generated by each cardiac cycle of the heart in real-time and is now widely used in heart rate detection (Homaeinezhad et al., 2012). This non-invasive detection method is easy to operate and has become an essential tool for assisting doctors in analyzing pathology. At this stage, the judgment of cardiovascular disease mainly depends on the doctor's experience. However, there are many types of heart diseases, and long-term manual detection makes it easy to cause false detection. How to quickly and accurately analyze specific diseases has become a new problem (Song et al., 2014). In addition, the traits of ECG signals include random, low-frequency, and susceptible, resulting in the diagnosis results are unstable. Intelligent automatic recognition and classification of ECG signals have become an inevitable choice to improve the efficiency and accuracy of ECG recognition (Yu and Chen, 2007).

With the maturity of Artificial Intelligence (AI) technology, many machine learning methods are used in the ECG signal feature detection, aiming at solving the problems related to large amounts of ECG signal feature data and a heavy load of manual detection. The typical methods are neural networks (NN) (Jiang and Kong, 2007), support vector machine (SVM) (Osowski et al., 2004), path forest (Luz et al., 2013), Independent Component Correlation Algorithm (ICA) (Sarfraz et al., 2014). Regarding neural networks, Jiang and Kong (2007) propose an evolutionary block-based neural network (BbNNs) for the classification of ECG signals. The BbNN is composed of a group of two-dimensional modular networks with flexible structure and internal configuration. The Hermite transform coefficient and the time interval between adjacent two R peaks are used as the input of BbNN. Osowski et al. (2004) introduce Hermite function as a feature extraction method in the SVM classifier, and also use higher-order statistics (HOS) to better extract features. Luz et al. (2013) introduced an optimal path forest classifier (OPF) to compare the performance of 6 distance metrics, six feature extraction algorithms, and three classifiers in two variants of the same data set. Although the accuracy of OPF is not as good as that of SVM, OPF is more efficient than SVM in terms of calculation time during training and testing phases. Sarfraz et al. (2014) use the basic function of a typical ECG signal obtained by ICA for pattern recognition. The features obtained by ICA are used as the training set. Although these methods have good performance and have achieved certain results, it is difficult to put into practice due to the severe deficiency of requiring manual design features.

In recent years, the machine learning and deep learning network has not only made remarkable achievements in the fields of image processing, audio recognition and many other fields (Wong et al., 2015a,b, 2016; Kandala et al., 2019; Pławiak et al., 2019, 2020), it has also been commonly used in the assisted diagnosis of heart disease based on ECG signals(Zubair et al., 2016; Acharya et al., 2017a,b; Yildirim et al., 2018; Gao et al., 2019; Atal and Singh, 2020; Pławiak and Acharya, 2020). Pławiak and Acharya (2020) use a deep genetic ensemble of classifiers to classify long-duration ECG signal (10 s). Gao et al. (2019) implemented an effective long short-term memory (LSTM) recurrence network model to classify 8 types of heartbeats. Atal and Singh (2020) proposed an optimization-based deep convolutional neural network to classify five different heartbeats. Compared with traditional neural networks, deep learning network can automatically extract features, recognize intricate data patterns, and eliminate complex signal preprocessing. Deep learning network also has a stronger nonlinear fitting ability, which has a better effect in identifying single-lead, multi-class, and unbalanced ECG datasets (Acharya et al., 2017a). Convolutional neural network (CNN) is a feedforward neural network that has been widely researched and used in deep learning, which has been applied successfully for the classification of arrhythmia (Zubair et al., 2016; Acharya et al., 2017a,b; Yildirim et al., 2018; Gao et al., 2019; Atal and Singh, 2020; Pławiak and Acharya, 2020) ECG signals. In the previous literature (Zubair et al., 2016; Acharya et al., 2017a,b; Yildirim et al., 2018; Atal and Singh, 2020), most of the works focus on the recognition of five main macro classes, namely Non-ectopic (N); Supraventricular ectopic (S); Ventricular ectopic (V); Fusion (F); Unknown (Q).

There is very little effort devoted to classify the micro-classes of the ECG signal, hence it serves as our main motivation to study the micro-classification heartbeats, of five types, i.e., Normal (NOR), Left Bundle Branch Block (LBBB), Right Bundle Branch Block (RBBB), Atrial Premature (AP), Premature Ventricular Contraction (PVC). The contribution of this paper is two-fold, in which the proposed algorithm is endowed with an ability to effectively process the non-filtered dataset with its potential anti-noise features, and secondly this paper presents an analysis of micro-classes of the ECG signal that compares some techniques of machine learning such as BP and Random Forest. The results can be served as a good source of benchmark literature to other researchers in the same field for future research work.

Section ECG Data Processing of the paper mainly introduces the ECG dataset used in this study and provides a detailed description of the data segmentation and preprocessing. In section Methodology, the architecture of the proposed algorithm and the specific experiment design is outlined. In section Result and Discussion, the performance and robustness of the proposed network is evaluated on the MIT-BIH Arrhythmia database and compared with BP, Random Forest, and several benchmarked CNN networks. Finally, section Conclusion summarizes the paper.

## ECG Data Processing

### ECG Dataset

The MIT-BIH database, an ECG database provided by the Massachusetts Institute of Technology and based on international standards and annotated information by multiple experts (Moody and Mark, 2001) is used in this study. The MIT-BIH database has been frequently used by the academic community in research for the detection and classification of arrhythmic heartbeats. The MIT-BIH database contains 48 ECG recordings, each recording time is 30 min, the sampling frequency is 360 Hz, and each ECG record is composed of two leads. MIT-BIH database can make adjustments and corrections based on the information annotated by experts and optimization algorithms. Furthermore, it learns from existing solutions for self-optimization.

### Pre-processing

**Figure 1:**
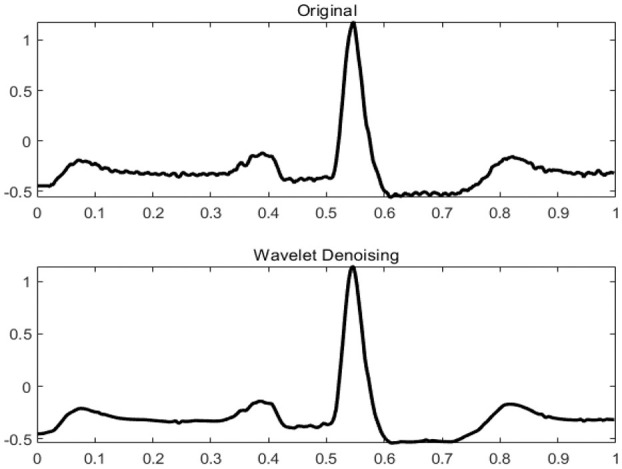
ECG signal before and after filtering.

ECG signals collected in a clinical environment are usually mixed with different interference, such as power frequency interference, baseline drift, and EMG interference. The raw data needs to be de-noised to make the classification more accurate. The bandpass filters, low-pass filters, wavelet transforms are widely used in the field of ECG denoising (Ahlstrom and Tompkins, 1985; Bazi et al., 2013; Wang et al., 2015; Yadav et al., 2015). In this paper, the wavelet transform method is used to preprocess the ECG signal. Wavelet transform is an algorithm that decomposes non-stationary signals into scale signals of different frequency bands. The filter uses an adaptive threshold filtering algorithm (Alfaouri and Daqrouq, 2008; Awal et al., 2014), and selects Sym4 in the Symlet wavelet function family as the wavelet function (Singh and Tiwari, 2006). Because the convolutional neural network has the feature of automatically extracting features from the inside of the signal, this paper only performs simple filtering on the signal, which can enhance the generalization of the network and reduce signal distortion. Figure 1 shows the ECG signal before and after filtering.

### Data Segmentation

In the MIT-BIH dataset, each heartbeat is marked with a disease annotation. This paper selects five heartbeats for classification, normal (NOR), left bundle branch block (LBBB), and right bundle branch block (RBBB), Atrial premature beats (AP), premature ventricular beats (PVC). The process starts by using the Pan-Tompkins algorithm to detected R-peak (Pan and Tompkins, 1985). Dataset is segmented into 360 samples and centered around the detected R-peaks. The process selects a single lead in the dataset, and all segments use the Z-score normalizing method (Acharya et al., 2017b).

### Data Enhancement

The unbalanced training set affects the feature learning of the convolutional neural network (Masko and Hensman, 2015), thereby reducing the recognition accuracy. This paper selects 16 recordings that contain most of these five heartbeats from the MIT-BIH dataset. After denoising and segmenting, oversampling is performed on the under-represented classes (Masko and Hensman, 2015). It randomly duplicates the under-represented classes, and discards the over-represented classes, which ultimately reduces the data imbalance of the training set. According to Table 1, only the C1 and C4 are enhanced because they greatly deviated from the average. The C2-C4 is not enhanced since they are only slightly imbalanced.

**Table 1:** Unbalanced and balanced dataset.

**Class**	**Classification dataset**		
	**Type**	**Unbalanced**	**Balanced**
C1	NOR	18,653	7,563
C2	LBBB	6,612	6,612
C3	RBBB	7,165	7,165
C4	AP	4,758	4,758
C5	PVC	1,208	6,324
**Total data**		38,396	32,422

### Ten-Fold Cross Validation

The original and de-noised data samples are used for experiments and 10-fold cross-validation is employed. This paper selects 16 recordings among the 48 recordings in the database, which contains nearly all of the five heartbeats that need to classify. A total of 32,422 heartbeats are extracted from 16 recordings, which are separated equally into 10 groups. 9 out of 10 groups are used in the training process while the remaining one of the remaining 10 groups is used for validation to get the optimal parameters. This process is iterated by 10 times by shifting test data. The performance is recorded after every iteration and integrated into one final confusion matrix at the end of the 10th iteration. The overall performance is calculated from the integrated confusion matrix.

## Methodology

### The Architecture

**Figure 2:**
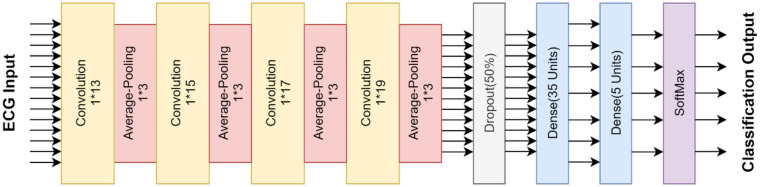
The architecture for the proposed CNN model.

This paper proposes a one-dimensional 12-layer convolution neural network (CNN) network structure to classify the five sub-classes of cardiac arrhythmia. CNN is a network consists of the input layer, convolution layer, pooling layer, fully-connected layer, and output layer. In contrast to traditional neural networks, CNN has convolution and pooling layers, which can extract and map features from input data to speed up learning and reduce over-fitting. Because the CNN has the feature of the multilayer perception, the two-dimensional convolution neural network has been widely used in image processing (Li et al., 2014; Wei et al., 2015). In this paper, we propose a one-dimensional 12 layer CNN to process a one-dimensional time series with uniform interval sampling (Kiranyaz et al., 2015). Several modifications are made in the network structure, i.e., the proposed CNN network uses the average-pooling layer instead of the max-pooling layer of the compared CNN network. The average-pooling layer can preserve the overall feature of the input data, which will be beneficial to classify the heartbeats. Also, the proposed CNN network has one more alternating convolution and pooling layer as compared to the benchmark CNN network. Table 2 summarizes the proposed CNN network architecture, including 8 alternating convolutions and average-pooling layers. They are followed by a dropout layer and two fully-connected layers, as seen in Figure 2.

- **Convolution layer**
  To process one-dimensional ECG signal, this paper uses a one-dimensional convolution kernel, which convolutes independently of the feature map of the previous layer. The output of the convolution layer is obtained by offsetting the convolution kernel and transferring it to the nonlinear activation function. The output expression is shown in Equation (1).
  (1) $$\begin{array}{l} h_{i}^{l,k}=f\left(b_{i}^{l,k}+\overset{N}{\underset{n=1}{\sum }}W_{n,i}^{l.k}\;*\;x_{i+n-1}^{l-1,k}\right) \end{array}$$
  Where, $h_{i}^{l,k}$ is the output of the ith neuron in layer *l*, *f*() is the activation function and $b_{i}^{l,k}$ is the offset of the neuron in layer *l*. $x_{i+n-1}^{l-1,k}$ is the output of neuron in layer *l-1*, $W_{n,i}^{l.k}$ is the *k*^*th*^ convolution kernels in *l*^*th*^ layer.
- **Pooling layer**
  Convolution of the next layer is commonly the pooling layer. By reducing the dimension of convolution layer output data, network complexity is reduced, as well as overfitting phenomenon. Robustness of the network is enhanced in this process. The pooling layer averages or maximizes the output features of the convolutional layer, and the corresponding methods are respectively, average pooling or maximum pooling. The output expression by Equation (2)
  (2) $$\begin{array}{l} o_{i}^{l,k}=f\left(\alpha_{i}^{l,k}pool\left(x_{i}^{l-1,k}\right)+b_{i}^{l,k}\right) \end{array}$$
  $o_{i}^{l,k}$ is the output of the *i*^*th*^ neuron in the *l* layer, *f*() is the activation function, $b_{i}^{l,k}$ is the offset of the neurons in *l* layer,$\alpha_{i}^{l,k}$ is the sampling weight coefficient, $x_{i}^{l-1,k}$ is the output of the neuron in *l-1* layer, *pool*() is the pooling function.
- **Fully-connected layer**
  After extracting features from multiple convolution layers and pooling layers, the fully-connected layer is used to expand the connection of all features. Finally, the SoftMax layer makes a logistic regression classification. Fully-connected layer transfers the weighted sum of the output of the previous layer to the activation function. The expression of the output is shown in Equation (3)
  (3) $$\begin{array}{l} o_{i}^{l,k}=f\left(w_{i}^{l,k}x_{i}^{l-1,k}+b_{i}^{l,k}\right) \end{array}$$
  $o_{i}^{l,k}$ is the output of the *l* layer of the *i*^*th*^ neuron, *f*() is the activation function, $b_{i}^{l,k}$ is the offset of the *l*^*th*^ layer of the neuron, $x_{i}^{l-1,k}$ is the output of the layer *l-1* of the neuron, $w_{i}^{l,k}$ is the network weight.
- **Dropout layer**
  There is usually a dropout layer before the fully-connected layer. The dropout layer will temporarily disconnect some neurons from the network according to the certain probability during the training of the convolution neural network, which reduces the joint adaptability between neuron nodes, reduces overfitting, and enhances the generalization ability of the network.
- **Training algorithm** The training algorithm of the convolution neural network is a backward propagation algorithm based on gradient descent. The network hyperparameters are estimated by the loss function, which is the deviations of the output vector and the expected output vector. Hyperparameters include the convolution kernel parameter *W* of the convolution layer, the sampling weight coefficient α of the pooling layer, the network weight *w* of the fully-connected layer and the offset *b* of each layer. The training of a convolution neural network consists of two phases, forward propagation and reverse propagation. In the forward propagation stage, the training data is input into the neural network, and the output vectors of the middle and output layers are calculated. In the reverse propagation stage, the output vectors of the output layer are compared with the expected output vectors and calculated the loss function with respect to the weights of the network. The loss is propagated back to the initial layers (in reverse direction) using the gradient descent method to update the weights for each neuron in every layer. Gradient descent comprises two steps: calculating gradients of the loss function, which is calculated by chain rules, then updating weight in the opposite or reverse direction of the gradient of the loss function, which is distinct from the forward calculation of loss function. A cost function is also calculated for the neuron output in each hidden layer to optimize the network hyperparameters continuously. The network ends training when it reaches the set error after multiple iterations.

**Table 2:** A summary table of the proposed CNN model for this work.

**Layers**	**Type**	**Output**	**Kernel size**	**Stride**
Layer 1	Convolution	360^*^16	1^*^13	1
Layer 2	Average-Pooling	179^*^16	1^*^3	2
Layer 3	Convolution	179^*^32	1^*^15	1
Layer 4	Average-Pooling	89^*^32	1^*^3	2
Layer 5	Convolution	89^*^64	1^*^17	1
Layer 6	Average-Pooling	44^*^64	1^*^3	2
Layer 7	Convolution	44^*^128	1^*^19	1
Layer 8	Average-Pooling	21^*^128	1^*^3	2
Layer 9	Dropout	21^*^128	-	-
Layer 10	Fully-connected	1^*^35	-	-
Layer 11	Fully-connected	1^*^5	-	-
Layer 12	SoftMax	1^*^5	-	-

Layer 1 is convoluted with the kernel size of 13 and the number of the filters of 16. An average-pooling layer with the size of 3 is applied, hence the output of layer 2 is reduced to 179^*^16. Then the feature map of layer 2 is convoluted with the kernel size of 15, and the number of the filter is 32 in layer 3. Again, an average-pooling layer is applied with the size of 3, reducing the neurons from 176^*^16 to 89^*^32 (layer 4). The convolution kernel size is 17, and the number of the filter is 64 in layer 5. An average-pooling layer with the size of 3 is applied after layer 5, reducing output to 44^*^64 (layer 6). The output of the layer 6 is convoluted with the kernel size of 19 and the filter number of 128 in layer 7. An average-pooling layer is applied afterward with a size of 3 (layer 8). Layer 9 is the dropout layer, which is set to 50%. Layer 10 is connected 35 neurons to layer 11. Finally, layer 11 connected 5 neurons to the SoftMax layer. A rectifier linear unit (ReLU) is used as an activation function before every average pooling layer. All fully-connected layer is applied with the L2 factor of 5 to reduce overfitting.

### Experiment Setup

In this paper, we ran a total of 60 epochs with a batch size of 36. The learn rate drop factor, learn rate drop period, and learning rate parameters are set to 0.1, 20, 10^−3^. The parameters of all proposed classifiers in Table 3 are selected for use based on the best results of ten-fold cross validation.

**Table 3:** All parameters of all proposed classifiers for this work.

**Network**	**Parameters**
1D-CNN	Epoch 60, MiniBatchSize 36, Optimizer adam, LearnRateDropFactor 0.1, LearnRateDropPeriod 20, InitialLearnRate 10e-3
Random Forest	NumberOfTree 500, MTry 18
BP	MaxEpoch 100, LearnRate 0.1, TrainingFunction trainrp, TrainingGoal 10e-3, Hidden node 20

Referring to Table 3, “MaxEpoch” is the maximum number of epochs to use for training. “MiniBatchSize” is the size of the mini-batch to use for each training iteration. A mini-batch is a subset of the training set that is used to evaluate the gradient of the loss function and update the weights. “InitialLearnRate” is the initial learning rate used for training. “LearnRateDropPeriod” is the number of epochs that passes between adjustments to the learning rate during training. “LearnRateDropFactor” is the multiplicative factor by which the learning rate drops during training. “Optimizer” is Adam (adaptive moment estimation) optimizer that we used. “NumberOfTree” is the number of trees that were used in random forest. “MTry” of the random forest is the number of features that were randomly selected in each split. The training function of the BP network is “trainrp,” which updates weight and bias values according to the resilient backpropagation algorithm. “TrainingGoal” is the mean squared error needing to reach at the end of the training. “Hidden node” is the number of neurons in the hidden layer.

In this paper, we construct BP neural network and Random Forest network for better comparison analysis, using the identical datasets. The BP network has 360 nodes in the input layer, correlated with 360 features of each sample has 20 neurons in the hidden layer, and 5 nodes in the output layer that represents five sub-classes; the largest value among the five neurons will be deemed as the classification output. On the other hand, the Random Forest network builds many decision trees and selects features randomly from random samples with bagging strategy, and uses the tress to vote for the input vector to get a class label. The input of each sample has 360 features, the number of the tree is set to 500, and 18 features are randomly selected to consider each split. The output is a five-class voter; the largest value will be taken as the classification output.

Furthermore, for fair comparison, we reconstruct a CNN network identical to Acharya et al. (2017b) to benchmark with our proposed algorithm (hereinafter referred to as the compared CNN network). To the best of our knowledge, there is no evidence in literature to study the micro-classification of heartbeats. Hence it is our biggest motivation to prove the viability of one-dimensional 12-layer CNN on the classifying the sub-classes of Arrhythmia by reconstructing other CNN network for better and fairer comparison.

### Evaluation Index

In order to evaluate and compare the classification effects of each model more accurately, this paper uses confusion matrix, accuracy (Acc), sensitivity (Sen), specificity (Spe), and positive prediction rate (Ppr) (Kiranyaz et al., 2015). Among them, the accuracy rate represents the ability to detect the real situation of the sample; the sensitivity represents the ability to distinguish various diseases; the specificity represents the ability to detect negatively for a certain disease; the positive predication represents the rate that proportion of positive identifications is actually correct. The corresponding expressions are formula (4–7):

(4) $$\begin{array}{l} Acc=\frac{TP+TN}{TP+TN+FP+FN} \end{array}$$

(5) $$\begin{array}{l} Sen=\frac{TP}{TP+FN} \end{array}$$

(6) $$\begin{array}{l} Spe=\frac{TN}{TN+FP} \end{array}$$

(7) $$\begin{array}{l} Ppr=\frac{TP}{TP+FP} \end{array}$$

Where TP stands for True Positive, TN stands for True Negative, FP stands for False Positive, FN stands for False Negative, FP stands for False Positive (Zubair et al., 2016).

## Result and Discussion

The proposed CNN algorithm is trained on a PC with Intel i5-7300HQ processors with 16GB of RAM and GTX1050 as GPU. It takes ~4236 s to complete training, and ~11 h to complete ten-fold cross validation. The average classification time of a single sample is 0.242 milliseconds. The implementation of the algorithm is using the MATLAB Deep Learning Toolbox and MATLAB Neural Network Toolbox.

The confusion matrix in Table 4 shows the classification results of the proposed CNN network. The overall classification accuracy rate of the five micro-class classification of heartbeats reaches 97.41%, and the positive rate and specificity of each category are over 90%, which has fully illustrated the effectiveness of the model. However, despite the data enhancement, the total number of C4 samples in the data set is still relatively small compared to the other four categories. Hence, the model's sensitivity to the C4 type is quite low.

**Table 4:** The confusion matrix of the proposed CNN network.

**The proposed CNN network**									
**Class**	**C1**	**C2**	**C3**	**C4**	**C5**	**Sen(%)**	**Spe(%)**	**Ppr(%)**	**Acc(%)**
C1	7,216	1	0	453	7	95.41	98.15	93.99	97.41
C2	0	6,609	0	2	0	99.96	99.99	99.97	
C3	1	0	7,149	2	2	99.78	99.98	99.93	
C4	333	2	2	4,297	3	90.31	98.77	92.67	
C5	13	0	14	4	6,312	99.81	99.88	99.51	

Tables 5–7 illustrate the confusion matrix of BP neural network, Random Forest network, and the compared CNN network, respectively. The result shows the accuracy rate of the proposed CNN algorithm is 97.41%, which is 10.16% higher than the BP neural network, 1.69% higher than the Random Forest, and 3.34% higher than the compared CNN network. Furthermore, the sensitivity and specificity of the proposed CNN network are higher than the other three networks. Compared with the traditional machine learning methods like BP network and the random forests, the CNN network has the weight-sharing feature, which significantly accelerates the optimization process. The proposed CNN network also shows better performance on extracting local features, which is essential to classify different heartbeat types. The proposed CNN network uses the average-pooling layer instead of the max-pooling layer of the compared CNN network. The average-pooling layer can preserve the overall feature of the input data, which will be beneficial to classify the ECG signal. Also, the proposed CNN network has one more alternating convolution and pooling layer than the compared CNN network. The filter size and number of each convolution layer are also larger than the compared CNN network.

**Table 5:** The confusion matrix of the BP network.

**BP network**									
**Class**	**C1**	**C2**	**C3**	**C4**	**C5**	**Sen(%)**	**Spe(%)**	**Ppr(%)**	**Acc(%)**
C1	6,573	79	14	1,580	252	86.91	92.25	77.34	87.25
C2	354	6,430	13	242	33	97.24	97.51	90.92	
C3	33	6	6,726	65	186	93.87	98.85	95.88	
C4	472	7	11	2,732	27	57.41	98.13	84.08	
C5	132	90	401	139	5,826	92.12	97.08	88.43	

**Table 6:** The confusion matrix of random forest.

**Random forest network**									
**Class**	**C1**	**C2**	**C3**	**C4**	**C5**	**Sen(%)**	**Spe(%)**	**Ppr(%)**	**Acc(%)**
C1	7,162	7	3	766	0	94.69	96.87	90.22	95.72
C2	6	6,577	2	24	3	99.47	99.86	99.47	
C3	11	12	7,044	24	0	98.31	99.81	99.33	
C4	364	10	8	3,934	3	82.68	98.60	91.08	
C5	20	6	108	10	6,318	99.90	99.44	97.77	

**Table 7:** The confusion matrix of the compared CNN network (Acharya et al., 2017b).

**Acharya's CNN network**									
**Class**	**C1**	**C2**	**C3**	**C4**	**C5**	**Sen(%)**	**Spe(%)**	**Ppr(%)**	**Acc(%)**
C1	6,873	11	3	1,043	9	90.87	95.71	86.73	94.07
C2	7	6,578	2	9	0	99.48	99.93	99.72	
C3	13	7	7,076	21	3	98.75	99.82	99.38	
C4	629	9	11	3,662	2	76.96	97.64	84.90	
C5	41	7	73	23	6,310	99.77	99.44	97.76	

Table 8 depicts the classification performance of the different networks applied to the original and denoising data sets. For the proposed CNN network, the accuracy rates of the original and de-noised data are 96.9 and 97.2%, respectively, and the accuracy rate of the classification of the original data is only decreased by 0.3%, which shows that the network proposed in this paper has a degree of noise resistance. The accuracy of BP neural network classification of raw data is dropped 3.3% compared to the de-noised data. The random forest is 0.5% lower, and the compared CNN network is also 0.5% lower, all of which are suffered more loss than the proposed CNN network.

**Table 8:** The performance of four different approaches.

**Network**	**Dataset**	**Acc(%)**	**Sen(%)**	**Spe(%)**	**Ppr(%)**
The proposed CNN	Denoising	97.41	97.05	99.35	97.21
	Raw	97.02	96.57	99.25	96.84
Random Forest	Denoising	95.72	95.01	98.92	95.58
	Raw	95.08	94.50	98.81	95.09
BP	Denoising	87.25	85.51	96.77	87.33
	Raw	85.98	84.94	96.46	85.14
Acharya et al. (2017b)	Denoising	94.07	93.17	98.51	93.67
	Raw	93.22	92.23	98.30	92.79

Table 9 lists the standard deviations of the sensitivities, specificities, and positive rates of four different networks. According to Table 9, the standard deviations of the four metrics of the proposed CNN network are less than the BP neural network, random forest, and the compared CNN network. The result indicates that the model's performance against multiple classifications is relatively stable, and the recognition effect of each classification is consistent, which shows the robustness of the model.

**Table 9:** The standard deviation of the four different networks.

**Network**	**Sen**	**Spe**	**Ppr**
The proposed CNN	3.79	0.75	3.20
Random Forest	6.43	1.18	4.07
BP	14.44	2.33	6.29
Acharya et al. (2017b)	8.75	1.62	6.49

Table 10 shows the existing literature of ECG classification. The dataset used in these literatures is not exactly the same, but the comparison is useful because classification is all on the same MIT-BIH database. Li and Zhou (2016) applied random forest classifier to recognize five main classes (N, Q, S, V, F), which achieved a 94.61% accuracy rate. Osowski et al. (2004) used an SVM classifier that achieved 98.18% accuracy on 13 classes of heartbeats. Martis et al. (2013) obtained 94.52% performance on five main classes (N, Q, S, V, F) in their studies. Pławiak and Acharya (2020) used a deep genetic ensemble of classifiers to classify long-duration ECG signal, which achieved 94.6% of accuracy on 17 arrhythmia classes in the MIT-BIH database. Gao et al. (2019) implemented an effective long short-term memory (LSTM) recurrence network model to classify 8 types of heartbeats (N, LBBB, RBBB, APC,NESC, ABERR, NPC, AESC). Atal and Singh (2020) proposed an optimization-based deep convolutional neural network, achieving 93.19% accuracy on five main classes (N, Q, S, V, F). Acharya et al. (2017a) achieved 95.22% of accuracy on the classification of two types of heartbeats only (Normal and MI) with an 11-layer CNN network. The architecture of this CNN network was 4 alternating convolutions and max-pooling layers, followed by 3 fully-connected layers. In another work, Acharya et al. (2017b) used 9-layer CNN network to classify 5 main classes (N, Q, S, V, F) and they achieved 94.03% of accuracy. For fair comparison, CNN model of Acharya et al. (2017b) is reconstructed to benchmark with the proposed model. Zubair et al. (2016) obtained a 92.70% classification performance rate for five main classes (N, Q, S, V, F) using an 8-layer CNN-based network. Zubair et al. (2016) implemented 3 alternating convolution and max-pooling layer in the CNN, followed by one MLP layer. Generally speaking, the proposed CNN network achieved relatively high accuracy on the 5 micro-classes of heartbeats classification.

**Table 10:** The performance comparison with other algorithms.

**References**	**No. of classes**	**Feature set**	**Classifier**	**Accuracy**
Li et al. (2014)	5	WPE + RR	RF	94.61%
Osowski et al. (2004)	13	HOS + Herminte	SVM	98.18%
Martis et al. (2013)	5	Cumulant + PCA	NN	94.52%
Acharya et al. (2017a)	2	End-to-end	CNN	95.22%
Acharya et al. (2017b)	5	End-to-end	CNN	94.03%
Zubair et al. (2016)	5	End-to-end	CNN	92.70%
Pławiak and Acharya (2020)	17	Frequency components	DGEC	94.60%
Atal and Singh (2020)	5	Gabor Filter + Wavelet	BaROA-DCNN	93.19%
The proposed CNN network	5	Wavalet	CNN	97.20%

Statistical variance test is carried out to study the differences of the classification performance of the proposed CNN algorithm as well as the different approaches for the raw and denoised dataset. Analysis of variance is a collection and representation of statistical model. The associated estimation procedures are used to analyze the differences among group means in a sample. The results are recorded in Tables 11–13. The *p*-value is the probability of obtaining test results at least as extreme as the actually observed results, under the assumption that the null hypothesis is correct. In the statistical significance test, the smaller the p-value, the stronger the evidence we should reject the null hypothesis. For analysis in Table 11, the null hypothesis is that the denoised data and raw data perform equally well. Significance level is set to 0.05. The *p*-value obtained from Table 11 shows 0.011 for accuracy, 0.006 for sensitivity, 0.009 for specialty and 0.027 for positive prediction rate. It clearly demonstrates all of the *p*-values are <0.05, therefore it has the full evidence of rejecting the null hypothesis. Thus, the effect of denoising is very significant in the classification of the micro-heartbeats type.

**Table 11:** Variance test analysis of the proposed CNN network.

**Class**	**Denoise data**	**Raw data**	**p-value**
Acc(%)	97.41 ± 0.27	97.02 ± 0.34	0.011
Sen(%)	97.05 ± 0.31	96.57 ± 0.38	0.006
Spe(%)	99.35 ± 0.07	99.25 ± 0.08	0.009
Ppr(%)	97.22 ± 0.31	96.84 ± 0.39	0.027

**Table 12:** Variance test analysis of different classifiers on raw data.

**Class**	**The proposed CNN**	**Acharya et al. (2017b)**	**Random forest**	**BP network**	***p*-value**
Acc(%)	97.02 ± 0.34	93.22 ± 0.49	95.08 ± 0.39	85.97 ± 0.57	0.0001
Sen(%)	96.57 ± 0.38	92.23 ± 0.50	94.50 ± 0.46	84.94 ± 0.62	0.0001
Spe(%)	99.25 ± 0.08	98.30 ± 0.12	98.81 ± 0.10	96.49 ± 0.14	0.0001
Ppr(%)	96.84 ± 0.39	92.81 ± 0.57	95.10 ± 0.40	85.14 ± 0.65	0.0001

**Table 13:** Variance test analysis of different classifiers on denoised data.

**Class**	**The proposed CNN**	**Acharya et al. (2017b)**	**Random forest**	**BP network**	***p*-value**
Acc(%)	97.41 ± 0.27	94.07 ± 0.26	95.72 ± 0.41	87.25 ± 0.60	0.0001
Sen(%)	97.05 ± 0.31	93.17 ± 0.25	95.01 ± 0.44	85.51 ± 0.65	0.0001
Spe(%)	99.35 ± 0.07	98.51 ± 0.06	98.92 ± 0.10	96.77 ± 0.15	0.0001
Ppr(%)	97.22 ± 0.31	93.68 ± 0.38	95.58 ± 0.45	87.34 ± 0.70	0.0001

On the other hand, in Table 12, the null hypothesis is under raw data, all four classifiers perform equally well. Significance level is still set to 0.05. The obtained *p*-values are too miniscule for accuracy, sensitivity, specialty, and positive prediction rate. All of the *p*-values are < 0.05, therefore it distinctly shows that the null hypothesis should be rejected. Thus, under raw data, it is obvious to interpret that the proposed CNN network performs much better than the rest of the classifiers.

In Table 13, the null hypothesis is under denoised data, all four classifiers perform equally well. Significance level is still set to 0.05. The obtained *p*-values are too miniscule for accuracy, sensitivity, specialty and positive prediction rate. All of the *p*-values are < 0.05, therefore it distinctly shows that the null hypothesis should be rejected. Thus, under denoised data, it is obvious to interpret that the proposed CNN network performs much better than the rest of the classifiers.

## Conclusion

Cardiovascular disease is a major health problem in today's world. The early diagnosis of cardiac arrhythmia highly relies on the ECG. Unfortunately, the expert level of medical resources is rare, visually identify the ECG signal is challenging and time-consuming. Different from the existing literatures in which most of them classify the five main classes, such as the Non-ectopic, Supraventricular ectopic, Ventricular ectopic, Fusion, and Unknown, in the MIT-BIH Arrhythmia database, our paper pays more attention to specific micro-classes, namely the Normal, Left Bundle Branch Block, Right Bundle Branch Block, Atrial Premature Beats, Premature Ventricular Beats. Compared with the BP neural network, random forests, and other CNN networks, it is worth to highlight that the proposed CNN network has relatively higher accuracy and robustness. The proposed CNN network shows an outstanding performance in the overall classification accuracy of 97.41%, sensitivity of 97.05%, specificity of 99.35%, and positive prediction rate of 97.21% on the classification of the micro-classes of Arrhythmia dataset.

The advantages of the proposed CNN network have been put to evidence. It is endowed with an ability to effectively process the non-filtered dataset with its potential anti-noise features. Besides that, ten-fold cross-validation is implemented in this work to further demonstrate the robustness of the network. In addition, this paper presents an analysis of the classification of micro-classes of the ECG signal with comparison to some techniques of machine learning such as BP and Random Forest. We add values to the research community by discussing the results of the classification of less popular micro-classes of Arrhythmia that can be served as a good source of benchmark literature to other researchers in this field for further research. One possible setback of the proposed solution is that it is computationally intensive to train the network, due to deep learning series is often attributed to large scale data required for training.

As for future work, it would be interesting to explore the use of optimization techniques to find a feasible design and solution. The limitation of our study is that we have yet to apply any optimization techniques to optimize the model parameters and we believe that with the implementation of the optimization, it will be able to further elevate the performance of the proposed solution to the next level.

## Data Availability Statement

Publicly available datasets were analyzed in this study. This data can be found here: https://physionet.org/content/mitdb/1.0.0/.

## Author Contributions

SYW: conceptualization, methods and materials, and supervision and review. MW and YL: data curation. MW, YL, and WY: formal analysis. WY: resources. MW: validation. MW, YL, WY, and SYW: writing. All authors contributed to the article and approved the submitted version.

## Conflict of Interest

The authors declare that the research was conducted in the absence of any commercial or financial relationships that could be construed as a potential conflict of interest.
